# Glymphatic Dysfunction Across Neurological Disorders: Links to Neurodegeneration, Inflammaging, Gut–Brain Signaling, and Physiological Modulators

**DOI:** 10.3390/biomedicines14102178

**Published:** 2026-09-25

**Authors:** Konstantinos I. Voumvourakis, Nikolaos S. Thomaidis, Eleni Sideri, Georgios N. Papadimitropoulos, Chrysa Liadinioti, Georgios Tsivgoulis, Sotirios Tsiodras

**Affiliations:** 1 2nd Department of Neurology, Faculty of Medicine, Attikon General Hospital, National and Kapodistrian University of Athens, Rimini 1, 12462 Athens, Greece; tsivgoulisgiorg@yahoo.gr; 2 2nd Department of Neurology, Metropolitan Hospital, 9 Ethn. Makariou & 1 El. Venizelou Str, 18547 Faliro, Greece; elenisideri1985@gmail.com (E.S.); ge.papadim@gmail.com (G.N.P.); chrysoulaliant@hotmail.com (C.L.); 3 Department of Chemistry, National and Kapodistrian University of Athens, Panepistimiopolis Zografou, 15771 Athens, Greece; ntho@chem.uoa.gr; 4 4th Department of Internal Medicine, School of Medicine, Attikon General Hospital, National and Kapodistrian University of Athens, Rimini 1, 12462 Athens, Greece; sotirios.tsiodras@gmail.com

**Keywords:** glymphatic system, neurodegenerative diseases, neuroinflammation, inflammaging, gut–brain axis, aquaporin-4, DTI-ALPS

## Abstract

The glymphatic system is a brain-wide perivascular network that facilitates cerebrospinal-fluid–interstitial-fluid exchange and the clearance of metabolic waste. Growing experimental and clinical evidence suggests that glymphatic dysfunction may represent a convergent pathophysiological feature associated with aging, neuroinflammation, and neurodegeneration, although its causal position within these processes remains unresolved. In this narrative review, we synthesize human imaging, clinical, and translational evidence implicating glymphatic dysfunction across major neurodegenerative diseases and integrate these findings with emerging data supporting its role in chronic low-grade inflammation associated with aging (“inflammaging”). We further discuss how alterations in the gut microenvironment may remotely influence glymphatic function and contribute to neurodegeneration through the gut–brain axis. Although supported by extensive preclinical evidence, studies in humans increasingly demonstrate impaired glymphatic function in several neurodegenerative disorders, most assessed using diffusion tensor imaging along the perivascular space (DTI-ALPS) index. Glymphatic dysfunction is associated with cognitive decline, motor impairment, and disease progression. Aging-related alterations in astrocytic function, aquaporin-4 polarization, blood–brain barrier integrity, and perivascular fluid dynamics provide mechanistic links between inflammaging and glymphatic failure. Gut dysbiosis may further exacerbate these processes by promoting central nervous system inflammation and vascular dysfunction. Together, these findings identify the glymphatic system as a clinically relevant pathway in neurodegeneration that may be regulated by aging-associated neuroinflammatory mechanisms involving the gut–brain axis. Finally, we discuss physiological, behavioral, and pharmacologic modulators that influence glymphatic function and propose a unified framework positioning glymphatic dysfunction as a central integrator of impaired brain clearance, with potential implications for biomarker development, clinical assessment, and disease-modifying therapeutic strategies in neurodegenerative disorders.

## 1. Introduction

Efficient clearance of metabolic waste and interstitial solutes is essential for maintaining central nervous system (CNS) homeostasis. Although the brain lacks a conventional lymphatic vasculature within its parenchyma, scientific findings in the last decade suggest the existence of a complicated mechanism, coined as the glymphatic system, which is responsible for the removal of macromolecules, neurotoxic proteins, and excess fluid [1,2]. With the characterization of the glymphatic system, our understanding of brain fluid dynamics has been fundamentally reshaped and a mechanistic framework with direct relevance to aging and neurodegeneration increasingly unveiled.

The glymphatic system is suggested to be a brain-wide perivascular network that facilitates the exchange between cerebrospinal fluid (CSF) and interstitial fluid (ISF), thereby enabling convective transport of solutes through the brain parenchyma [3]. According to the prevailing theory, CSF enters the brain along periarterial spaces, driven by arterial pulsatility and pressure gradients, mixing with ISF within the interstitium [4]. The astrocytic endfeet ensheathing the cerebral vasculature is considered a key structural element of glymphatic efficiency, forming the outer boundary of perivascular spaces, expressing aquaporin-4 (AQP4) water channels that enable glymphatic flow. Proper polarization of AQP4 channels at the perivascular astrocytic endfeet is of crucial importance for efficient waste clearance [5]. After traversing the interstitial compartment, fluid and solutes exit the brain along perivenous spaces, ultimately draining toward meningeal lymphatic vessels and cervical lymph nodes [3]. Through this pathway, the glymphatic system is suggested to contribute to the clearance of neurotoxic proteins, including amyloid-β (Aβ) [1], tau [6], and α-synuclein (SNCA) [7] (Figure 1).

Aging has been proposed to exert a profound impact on glymphatic structure, as seen in the loss of perivascular AQP4 polarization [8], vascular stiffness [9], and altered CSF dynamics [10]. Glymphatic function is also altered with advancing age [11], possibly due to reductions in arterial pulsatility [12] and astrocytic reactivity [13]. Chronic low-grade immune activation, also known as inflammaging, further contributes to these changes.

Gut dysbiosis leads to increased expression of pro-inflammatory cytokines resulting in systemic inflammation. Inflammatory mediators from the gut may enter the CNS and impair glymphatic function [14]. Moreover, the production of neurotransmitters and microbial metabolites from the gut may interfere with the circadian rhythms, thus leading to sleep disturbances [15]. Given the positive impact of sleep on glymphatic activity [4], any sleep disturbance or disorder may lead to the accumulation of neurotoxic waste in the brain, potentially contributing to the excessive neuroinflammation that is present in major CNS disorders.

Given its proposed role in clearing aggregation-prone proteins and inflammatory mediators, there is increasing evidence highlighting the potential implication of the glymphatic system in neurodegeneration as a convergent mechanism in multiple CNS disorders [16,17,18,19,20,21,22,23]. Although direct monitoring of glymphatic flow in humans remains challenging, several in vivo imaging approaches have been developed to infer glymphatic function [24]. Contrast-enhanced and contrast-free magnetic resonance imaging (MRI) are among the in vivo techniques that can be applied to humans. The former is invasive and requires the injection of a paramagnetic contrast agent, while the latter analyzes CSF movements without the need for a tracer. Consequently, contrast-free MRI techniques have become the primary tools in human studies, with diffusion tensor imaging along the perivascular space (DTI-ALPS) being the most widely used technique. DTI-ALPS quantifies directional water diffusivity along perivascular spaces, particularly in the white matter, providing an indirect index of perivascular fluid dynamics. Importantly, however, DTI-ALPS does not directly measure CSF–ISF exchange, solute clearance, or bulk glymphatic flow. The index is influenced by the underlying white matter microstructure [25], acquisition parameters, age, and cerebrovascular pathology [26]. Accordingly, altered DTI-ALPS should be interpreted as altered perivascular diffusivity potentially consistent with altered glymphatic function, rather than as a direct quantitative measurement of whole-brain glymphatic clearance [27]. Regardless of these limitations, the DTI-ALPS index has been widely employed in a plethora of studies tackling neurodegenerative disorders [28]. Additional MRI-based markers monitoring enlarged perivascular space (ePVS) burden [29] and choroid plexus volume [30] are used separately or in combination with the DTI-ALPS index.

The scope of this review is to synthesize existing preclinical and translational evidence for glymphatic system dysfunction in neurodegenerative diseases. The last section is dedicated to the discussion of the physiological regulators of glymphatic activity. We build upon this synthesis while we integrate emerging concepts of inflammaging and gut–brain-axis interactions. Our goal is to highlight the possibility of a systemic nature of the glymphatic system that may contribute to the onset of CNS disorders. We additionally aim to underscore the importance of the clinical assessment of waste clearance mechanisms in developing useful insights for future diagnostic and therapeutic strategies.

## 2. Literature Search Strategy

The present work has been prepared as a narrative review. The analysis strategy was informed by a structured literature search of PubMed/MEDLINE conducted through the end of December 2026. Applied search terms combined concepts related to the glymphatic system and function (“glymphatic”, “glymphatic clearance”, “perivascular space”, “DTI-ALPS”, “aquaporin-4”, “CSF–ISF exchange”) with terms describing neurodegenerative and neuroinflammatory disorders, aging/inflammaging, the gut–brain axis, and physiological or pharmacological modulators. Original experimental and human studies were primarily considered, while relevant systematic and narrative reviews were used to identify additional primary literature. Priority was given to studies directly evaluating glymphatic-related fluid transport, tracer clearance, AQP4 localization, DTI-ALPS index, size of perivascular spaces, or other imaging markers relevant to brain clearance. Articles lacking direct relevance to CNS fluid or clearance pathways, as well as studies presenting hypothetical models or containing non-informative secondary reports, were excluded.

## 3. Glymphatic Dysfunction Across Neurological Disorders

### 3.1. Neurodegenerative Disorders

#### 3.1.1. Alzheimer’s Disease (AD)

Expression of AQP4 has been reported to correlate with advancing age, while loss of its perivascular localization is associated with increased amyloid burden and higher Braak stage [31]. In an experimental model of AD (5XFAD mice) where AQP4 was coupled with a-syntrophin (Snta1), deletion of the latter allowed for controlled depolarization of AQP4 without alterations in its expression [32]. Both deficiency and mislocalization of AQP4 resulted in increased parenchymal Aβ plaque formation and depositions in *Snta* mice compared to their littermates. Furthermore, human postmortem case series analysis demonstrated that the loss of perivascular localization of AQP4 in cortical gray matter was more evident in patients with AD compared to subjects with earlier stages of cognitive decline [33]. Genetic analysis has further unveiled *AQP4* polymorphisms that are associated with higher risk of cognitive decline [34] and modification in Aβ uptake [35]. However, other studies have reported a protective role of AQP4 deficiency leading to mitigated astrogliosis [36], neuronal apoptosis, and uptake of Aβ [37].

Altered glymphatic flow has been reported following depletion or pharmacological inhibition of AQP4 in the Aβ precursor protein/presenilin 1 model (APP/PS1) [36], the 5X FAD model [38], and tauopathy models [6,18,39]. In all instances, impaired clearance was associated with toxic accumulation of Aβ and tau, astroglial atrophy, loss of synaptic proteins [36], memory deficits, behavioral impairment, and neuronal dysfunction [38]. Suppression of clearance of toxic waste following deletion of *AQP4* is further supported by the slowing down of CSF influx by almost 70% [1]. Particularly for models of tauopathy, impairment of glymphatic flow seems to take place in specific brain regions, such as the rostral cortex, which are affected early during tau deposition [18]. Glymphatic flow stagnation in these areas is associated with increased volume changes that are potentially implicated in neurodegeneration and diminished cognitive performance [6,39].

Experimental results of *AQP4* deficiency widely coincide with findings from studies assessing the effects of meningeal lymphatic disruption that are involved in the drainage of CSF and ISF to cervical lymph nodes. Surgically disrupted meningeal lymphatics in AD mouse models increased Aβ deposition both in the meninges and the brain tissue, accompanied by increased inflammation and behavioral impairment [40]. Disrupted meningeal lymphatics were further correlated with a pro-inflammatory microglial molecular signature like that observed in AD pathogenesis [41]. Given that the dominating microglial phenotype is suggested to be the key mediator for the association between tauopathy and glymphatic dysfunction on the one hand [42,43], and that Aβ accumulation in the CSF may halt the glymphatic influx on the other hand [44], a waste clearance mechanism dysfunction driven by pathological features observed in AD pathogenesis is further reinforced.

Advanced imaging studies have revealed consistent clinical symptoms associated with glymphatic dysfunction across the AD spectrum. These studies aimed to link glymphatic-related imaging abnormalities to core AD pathology comparing DTI-ALPS values in patients with AD and patients with prodromal stages of AD pathogenesis, such as patients with mild cognitive impairment (MCI) and subjective cognitive decline (SCD), as well as healthy individuals [16,20,45,46,47,48]. DTI-ALPS alterations were reported in symptomatic AD compared to healthy individuals [45]. Altered DTI-ALPS is already detectable in SCD, suggesting that changes in perivascular diffusivity may represent an early imaging marker associated with preclinical AD-related processes [20], which is supported by PVS enlargement even before perivenous efflux impairment [19]. Lower DTI-ALPS values were strongly associated with greater amyloid burden [19,20,45,46] and reduced cognitive function [19,20,45]. Interestingly, Aβ and tau burden partially or fully mediate the association of low DTI-ALPS index with progression from prodromal stages to an AD phenotype [16,46] and deterioration of cognitive performance [20,46]. Lower DTI-ALPS index is further associated with enlarged PVS [48], while free water in white matter was correlated with reduced scores in cognitive and functional scales [19]. No significant risk associations were observed when patients with MCI were stratified by sex, age, or *APOE ε4* status [16] (Supplementary Table S1).

These findings suggest a clinically meaningful association between reduced water diffusivity and neuronal loss, potentially linking glymphatic dysfunction to AD pathology. Glymphatic dysfunction in AD is further supported by a two-stage proteomic analysis in patients with AD and MCI that shows that proteins associated with the proper function of glymphatic system are mainly involved in neurodegenerative processes, immunity, and inflammation [49].

#### 3.1.2. Parkinson’s Disease (PD)

Deficiency of *AQP4* in established PD experimental models has been shown to drive hypersensitive responses in mice following stimulation with neurotoxic agents [50,51]. These reactions were suggested to be due to increased astrogliosis, since an increased number of astrocytes was observed following *AQP4* deficiency in basal as well as neurotoxic conditions [52]. Genetic evidence from PD patients further supports the role of AQP4 in the disease pathogenesis, with patients carrying the *AQP4 rs68006382* being associated with faster transition to lower cognitive performance [53].

Reduced clearance of SNCA has been observed following deletion or pharmacological inhibition of AQP4 in experimental models overexpressing the human A53T-SNCA [7]. Accumulated SNCA was associated with neuronal loss and exacerbated symptoms. Additionally, in PD models where SNCA preformed fibrils (PFFs) were injected intrastriatally, glymphatic flow stagnation was associated with behavioral impairments [54]. Furthermore, blockage of meningeal lymphatic drainage, via cervical lymph node ligation, resulted in impaired clearance of SNCA and depolarization of AQP4, further highlighting the impact of the clearance mechanisms in PD pathogenesis [55].

A direct feedback loop between glymphatic function impairment and SNCA aggregation has been proposed. Overexpression of A53T-SNCA in mice has been shown to suppress glymphatic flow via modulation of the expression and depolarization of AQP4 [7]. Subsequent blockage of PVS by SNCA aggregates may not further permit the glymphatic efflux. Alternatively, targeted expression of mutated human SNCA in astrocytes drives the loss of AQP4 perivascular localization associated with enhanced astrogliosis in the brainstem and spinal cord of symptomatic mice, potentially linking neuroinflammation and glymphatic system dysfunction [56].

DTI-ALPS alterations have been consistently reported in PD patients compared to matched healthy individuals across cross-sectional and longitudinal cohorts [23,57,58,59,60]. Lower DTI-ALPS index is associated with greater motor severity, as measured by higher Unified Parkinson’s Disease Rating Scale (UPDRS)-III scores [58,60,61], whereas baseline glymphatic-related abnormalities may predict motor dysfunction progression, with lower DTI-ALPS associated with faster worsening of UPDRS-III scores [23] and increased risk of clinical milestones, such as wheelchair dependence [62]. Furthermore, lower DTI-ALPS is associated with worse global cognition, as measured by lower scores in the Mini-Mental State Examination (MMSE) and Montreal Cognitive Assessment (MoCA) scales in several PD cohorts [60,61], while baseline DTI-ALPS values can predict longitudinal cognitive decline [60]. However, findings from other PD cohorts have not been able to replicate this association [23].

Furthermore, lower DTI-ALPS values seem to decline with advancing age, with older PD subgroups demonstrating stronger negative correlations [23]. Increased age at disease onset is also associated with decreased DTI-ALPS values, suggesting that aging-related glymphatic alteration may be associated with disease onset [59]. Moreover, impaired clearance has been linked with sleep disturbances [61,63] possibly associated with prodromal PD phenotypes, although this association is emerging more clearly in clinically meaningful PD subgroups [61]. Female patients have shown higher DTI-ALPS values compared to males at baseline and after one year of follow-up [23]; however, other studies do not confirm sex-specific differences [61] (Supplementary Table S1).

A potential direct link between glymphatic system impairment and the subsequent neurodegeneration in PD patients may be theorized based on indications about a positive association of the DTI-ALPS index with regional cortical volumes [57]. Mediation analysis demonstrated that cortical degeneration partially mediates the association between glymphatic dysfunction and cognitive impairment. Given that accumulation of SNCA is most often detected in the regional cortex, this evidence suggests a bidirectional mechanism through which SNCA aggregates may disturb the AQP4 anchoring in the astrocytic endfeet, resulting in glymphatic clearance abolition, further promoting its own aggregation.

#### 3.1.3. Frontotemporal Dementia (FTD)/Non-AD Dementia

Categories of non-AD dementia encompass a variety of conditions that cause cognitive decline, distinct from AD. Among several conditions, such as frontotemporal dementia (FTD) and idiopathic normal pressure hydrocephalus (iNPH), the occurrence of glymphatic system dysfunction is suggested. The FTD group characterizes brain disorders that primarily affect the frontal and temporal lobes, with the most common clinical syndrome being the behavioral variant *FTD (bvFTD)* which is associated with atrophy of the frontal and anterior temporal lobes [64]. The pathology of FTD is characterized by intracytoplasmic inclusions of aggregated neuronal and glial proteins, such as tau and TDP-43, that eventually lead to disruption of neuronal homeostasis and subsequent cell death [65,66]. On the other hand, iNPH pathogenesis is associated with altered CSF dynamics, enlarged brain ventricles, and vascular abnormalities [67].

Experimental findings from studies employing vascular/mixed-dementia experimental models including the microscopic microinfarcts model [68], the hyperhomocysteinemia model of vascular cognitive impairment [69], and a mixed dementia rat model, namely using SHRSP/FAD rats [70], point towards a systemic clearance impairment. The polarization of AQP4 was found to be disturbed in all experimental models, while associations with cognitive decline [69], reactive gliosis [71], dilated PVS, and delayed waste clearance [68] were observed. These initial findings suggest that glymphatic system dysregulation may emerge as a unifying contributor across diverse dementia types.

Significantly higher levels of AQP4 have been reported in FTD patients compared to subjects not affected by neurodegenerative diseases. A significant positive correlation was further detected between AQP4 and total tau levels, pointing towards enhanced astrogliosis [72]. Conversely, decreased expression [73] and reduced density of AQP4 in astrocytic endfoot membranes has been reported in biopsies of iNPH patients compared to reference individuals [74].

Altered DTI-ALPS values have been demonstrated in patients with FTD syndromes and genetic FTD [22,75,76]. Particularly for bvFTD, increased waste accumulation suggestive of glymphatic abnormality has been reported in characteristic areas of FTD pathology, such as the forebrain and midbrain areas [75]. Across FTD variants, DTI-ALPS abnormalities are subtype-specific and are related to whole-brain white-matter hyperintensities, cognitive performance, and cortical thickness, suggesting that impaired clearance is linked to specific patterns of cortical neurodegeneration [76]. Furthermore, assessment of solute clearance across symptomatic/presymptomatic mutation carriers and non-carriers demonstrated that the DTI-ALPS index was lower in symptomatic compared to presymptomatic patients and non-carriers [22]. The index was further associated with disease severity and more rapid worsening, suggesting that impaired clearance may predispose the disease course based on the different genetic background and disease stage [22].

Similar findings have been reported for cohorts of patients with iNPH. Clearance of gadobutrol has been reported to be delayed in iNPH patients compared to healthy individuals, showing increased persistence in the brain parenchyma [77,78,79]. Compared with non-cognitively affected patients, iNPH patients show higher medial temporal lobe atrophy scores and ventricular enlargement (Evans’ index) and reduced entorhinal cortex thickness, tying impaired clearance with structural vulnerability in memory-related regions [79]. These observations suggest that reduced clearance of toxic metabolites may underly pathology in non-AD dementia (Supplementary Table S1).

#### 3.1.4. Amyotrophic Lateral Sclerosis (ALS)

Studies in transgenic animals expressing mutated human superoxide dismutase (SOD) 1, which mimics ALS pathogenesis, have revealed broad changes in astrocyte homeostasis; deregulated expression of AQP4 was accompanied by depolarization [80]. These pathological features were suggested to have contributed to earlier disease onset [81], potentially due to altered CSF dynamics [82]. Glymphatic impairment during early stages of ALS is further supported by a study in transgenic mice expressing cytoplasmic human TDP-43 (hTDP-43ΔNLS) under the control of doxycycline [83]. Following the cessation of doxycycline feeding, clearance of an MR agent (Magnevist) was evaluated using dynamic contrast-enhanced MRI. Altered glymphatic function was observed in transgenic mice three weeks after doxycycline withdrawal, indicating disruption at a relatively early stage in the neurodegenerative process, estimated to begin around four weeks. Subsequently, transgenic mice exhibited signs of progressive neurodegeneration in the primary motor cortex, the primary somatosensory cortex, and the corticospinal tract [83].

Across recent human ALS studies, glymphatic flow has been assessed using non-contrast MRI biomarkers, most commonly DTI-ALPS and estimation of choroid plexus volume fraction. Multiple datasets demonstrate lower DTI-ALPS index in patients with ALS compared to age- and sex-matched healthy individuals [84,85,86,87] and patients with other motor neuron diseases [88], consistent with impaired perivascular water diffusivity. A longitudinal study confirmed that glymphatic impairment could be a persistent alteration detectable during follow-up of up to 8 months [88]. Interestingly, similar to stroke, unilateral lowered DTI-ALPS index has been reported, with right-side function being significantly reduced in ALS patients compared to healthy individuals, while the left side was not affected [86]. These observations suggest that glymphatic impairment may contribute substantially to neuronal damage in ALS in a unique manner.

Clinical and demographic factors affect waste clearance in ALS patients. The DTI-ALPS index has been shown to decline with advancing age [86,88], while choroid plexus volume fraction is also age-associated [85], potentially linking aging with vulnerability to ALS pathology due to gradually impaired perivascular diffusivity. Alternatively, increased choroid plexus volumes may represent compensatory CSF production due to impaired waste clearance [85]. Moreover, female ALS patients were shown to have higher mean DTI-ALPS values compared to their male counterparts [86]. Positive association with metrics of clinical disability [87,88] reflecting sensorimotor dysfunction and disease duration [84] has also been reported, suggesting a link between glymphatic and functional neural impairment with disease severity. However, other studies found no correlation with King’s stage, cognitive function questionnaires, and other clinical parameters [85,86]. Lastly, impaired clearance in ALS patients has been associated with sleep disturbances [87] (Supplementary Table S1).

Collectively, glymphatic impairment in early stages of ALS pathogenesis may substantially contribute to the disease burden. Therefore, clinical assessment of glymphatic function could potentially provide cues for designing a better therapeutic approach earlier in the disease course.

### 3.2. Neuroinflammatory Disorders

#### 3.2.1. Multiple Sclerosis (MS)

Increased expression and loss of perivascular localization of AQP4 have been shown in the brain and spinal cord tissues in the most commonly used experimental model of MS, experimental autoimmune encephalomyelitis (EAE) [89,90,91]. Deficiency of *AQP4* in myelin oligodendrocyte glycoprotein (MOG)-induced EAE, as well as in adoptive transfer models, was associated with mitigated neuroinflammation and diminished clinical symptoms compared to wild-type littermates [92]. Moreover, following adenovirus-mediated overexpression of AQP4 in astrocytes, the latter acquired an enhanced pro-inflammatory profile, further reinforcing the role of AQP4 in mediating the MS inflammatory cascade [92].

Widespread parenchymal localization of AQP4 is associated with impairment of parenchymal CSF circulation in the spinal cord of EAE-induced mice [91]. Although deceleration of flow could plausibly be based on the heavy inflammatory burden of MS or the hypersecretion of CSF [93], an increasing body of research points towards glymphatic system dysfunction in patients with MS. DTI-ALPS values have been consistently reported to be reduced in cohorts of MS patients compared to age- and sex-matched healthy individuals [17,94]. Although glymphatic deregulation is apparent both in the relapsing–remitting and the progressive phenotype of MS, an enhanced decline is observed for the latter, suggestive of a parallel trajectory along clinical course [95]. Lower DTI-ALPS index is further associated with worse clinical disability, as estimated with the Expanded Disability Status Scale, and longer disease duration [17,95].

Impaired clearance seems to co-occur with structural brain damage and global brain volume changes. Lower DTI-ALPS index was reported to be associated with higher white-matter and cortical lesion volume, deep grey matter atrophy, and reduced fractional anisotropy [17]. Additionally, larger choroid plexus volume correlates with lower DTI-ALPS index in patients with MS, while both indices were associated with higher total, periventricular, and juxtacortical white-matter lesion volumes and lower brain, thalamic, and cortical volumes [94]. Interestingly, the DTI-ALPS index was reported to partially mediate the association between increased choroid plexus volume and structural brain changes, suggesting that glymphatic impairment links altered CSF dynamics to tissue damage [94] (Supplementary Table S1).

Choroid plexus enlargement may represent a key factor in glymphatic system dysfunction in MS, potentially driven by increased infiltration of inflammatory cells and soluble mediators [96,97]. Given that choroid plexus is suggested to produce the vast majority of CSF, any structural distortion may potentially lead to stagnation of CSF flow [30]. Given that choroid plexus enlargement takes place during the early stages of MS, correlations with increased presence of immune cells [98], clinical disability, and cognitive dysfunction [99] point towards a more progressive phenotype [30].

Collectively, MS-specific aberrations in choroid plexus volume may confer persistent inflammation that may lead to the impediment of glymphatic flow and subsequent impairment of the clearance of immune toxic mediators [94]. Impaired glymphatic clearance is reported to be associated with both demyelinating and neurodegenerative events, and therefore the exact impact of glymphatic system deregulation in MS pathogenesis warrants further investigation.

#### 3.2.2. Autoimmune Encephalitis

Autoimmune encephalitis is an umbrella term for multiple disorders that are characterized by the immune system activation against CNS epitopes such as N-methyl-D-aspartate receptor [100]. Concrete findings regarding the expression of AQP4 in autoimmune encephalitis are lacking, although recently the rare coexistence of autoantibodies against neuronal or glial epitopes representative of autoimmune encephalitis and against AQP4 has been reported [101]. This clearly distinguishes the disease mechanisms of autoimmune encephalitis from neuromyelitis optica spectrum disorder.

Only one recent study has evaluated the glymphatic function in patients with autoimmune encephalitis [102]. Patients with autoimmune encephalitis had lower DTI-ALPS values compared to age- and sex-matched healthy individuals. A low baseline DTI-ALPS index was associated with a decline in cognitive function, as estimated with MMSE and MOCA scales, while an increased risk of long-term cognitive impairment was linked to lower DTI-ALPS index. These observations were confirmed across different subgroups depending on the target of the autoimmune pathogenesis, while the increased expression of pro-inflammatory cytokines partially mediate the association of glymphatic function deterioration with cognitive decline [102] (Supplementary Table S1). Although preliminary, these observations suggest a link between cognitive decline and inefficient clearance of pro-inflammatory cytokines.

### 3.3. Acute/Acquired CNS Injury

#### 3.3.1. Stroke

The presence of abundant toxic extracellular fluids in areas of liquefactive necrosis leads to atrophy, cytotoxic edema, and neuronal loss in regions adjacent to the infarct for weeks following stroke [103]. Therefore, potential dysregulation of waste clearance mechanisms during stroke may contribute to impartial recovery. The glymphatic flow could plausibly be hampered by the rapid inflow of blood and CSF in the PVS, resulting in brain edema and tissue swelling [104,105,106]. Evidence points to increased enlarged-PVS burden, particularly in the basal ganglia and hippocampus, as a significant risk factor of incident stroke and intracerebral hemorrhage (ICH) [107,108]. Additionally, patients with spontaneous ICH and increased size of PVS in the centrum semiovale demonstrate increased vascular amyloid burden [109], while in patients with subarachnoid hemorrhage (SAH), a reduced serum/CSF ratio of neurofilament light polypeptide has been observed [110]. These findings point towards impaired glymphatic efflux and reduced leakage to the draining lymph nodes.

The expression of AQP4 in SAH, ICH, and ischemic stroke experimental models has been reported to increase very early during induction [111], suggesting its crucial role in mediating glymphatic function integrity. However, it is difficult to define the role of AQP4 in stroke and related conditions due to the contradictory results reported following its deficiency in experimental models. Knockout of *AQP4* has been shown to cause enhanced neuronal loss and BBB disorganization following SAH induction [105,112] and brain edema [113], as well as pronounced microglial activation in the middle cerebral artery occlusion (MCAO) model compared to wild-type mice [114]. On the other hand, cerebral edema is reported to decrease in *AQP4* knockout mice early post-MCAO-induction [115,116], while reduced infarct volumes following APQ4 inhibition have been also reported [117]. Therefore, defining the role of AQP4, and subsequently the regulation of glymphatic flow in stroke, warrants further research.

However, experimental evidence suggests that impaired clearance occurs during stroke and related conditions. Rodent models of SAH, whether induced by endovascular perforation or blood injection into the cisterna magna, are characterized by a significant and prolonged disruption of CSF circulation and glymphatic flow [105,118,119,120] as well as reduced drainage to deep cervical lymph nodes associated with AQP4 depolarization [118]. Findings from a non-human primate model of SAH further corroborate the dysregulation found in rodent models [121]. Glymphatic system dysfunction during the acute phase of multiple experimental models seems to predominantly occur in the substantia nigra and the ventral thalamic nucleus (VTN) [122]. Deregulation of glymphatic flow has been associated with accumulation of tau protein and immune cells, glial cell activation, neuroinflammation, neuronal apoptosis [118], and cognitive dysfunction [123], reflecting extended neuropathological damage.

Accumulating neuroimaging evidence indicates that waste clearance is consistently impaired in stroke patients, most inferred from reductions in the DTI-ALPS index. Across ischemic and hemorrhagic stroke subtypes, patients demonstrate lower DTI-ALPS values compared to healthy individuals [124,125,126,127,128,129] and disease-specific subgroups [21], supporting the presence of glymphatic dysfunction post-stroke.

In patients with subacute ischemic stroke, significantly lower DTI-ALPS values compared to healthy subjects indicate reduced perivascular fluid transport after ischemic injury [126]. During the chronic phase (3–12 months), stroke survivors demonstrated persistent reduced DTI-ALPS values compared to healthy subjects, suggesting prolonged glymphatic disturbance beyond the acute/subacute period [128]. This observation supports the existence of long-lasting glymphatic impairment, particularly in the hemisphere ipsilateral to infarction [125,126,128,130]. Similar, glymphatic-related alterations have been demonstrated in the lesion side compared to the contralateral side in patients with spontaneous ICH [124]. Patients with cerebral infraction classified in a good-prognosis group based on modified Rankin scale had increased DTI-ALPS values compared to those classified in the poor-prognosis group [21]. Preserved glymphatic function was associated with better neurological outcomes and better psychological recovery, pointing towards a prognostic value of DTI-ALPS [21]. Particularly for patients with cerebral small vessel disease (CSVD), a condition that causes approximately 20% of strokes [131], reduction in DTI-ALPS indices correlated with enlarged PVS in basal ganglia and existence of lacunes [132], cerebral venous integrity [133], and vascular risk factors [129] (Supplementary Table S1).

Findings from these studies suggest an association between impaired clearance and motor and cognitive functions. In subacute ischemic stroke, the DTI-ALPS index correlated positively with Fugl-Meyer motor scores, supporting a relationship between greater glymphatic impairment and worse motor function [126]. Furthermore, the DTI-ALPS index was correlated with 6-month post-stroke cognitive impairment [125]. In concordance with this, the DTI-ALPS index has been reported to be useful for predicting future dementia risk in patients with lacunar strokes and confluent white-matter hyperintensities [134]. Weak correlations also emerged at 3 months between the DTI-ALPS index in the lesioned hemisphere and motor and memory scales, although this correlation could not be detected at 1 year of follow-up, suggesting that the correlation may be transient [128] (Supplementary Table S1).

The time since stroke onset and stroke duration are among the factors that suggest a time-dependent relationship between glymphatic activity and disease course. Values of DTI-ALPS increase along with time since disease onset [125,128,130], while in the sICH cohort, the lesion-side DTI-ALPS index was correlated with disease duration [124]. These observations may reflect the activation of organism towards partial recovery after early impairment due to potential remodeling or reversibility of glymphatic function during rehabilitation [125,130]. Application of standard reperfusion therapy results also in partial recovery of waste clearance [135], further reinforcing its importance in the therapeutic landscape of stroke.

#### 3.3.2. Traumatic Brain Injury (TBI)

Widespread and persistent disruption of waste clearance has been demonstrated in several experimental models of TBI, characterized by impaired CSF influx and efflux very shortly post-injury [136]. This disruption could endure up to 10 weeks in adult mild TBI models, particularly in areas such as the olfactory bulb [137,138]. Disruption of normal brain efflux kinetics is further supported by the reduced drainage to the cervical lymph nodes [136]. Glymphatic impairment was linked to loss of motor coordination and balance deficits. Mechanistically, increased noradrenaline levels released due to post-traumatic sympathetic overactivation seem to contribute to the stagnation of glymphatic flow by suppressing vessel contractility, leading to edema and impaired debris clearance [139].

Deregulation of clearance has been associated with alterations in AQP4 expression and polarization [140,141]. However, the role of AQP4 seems to be quite complex. Reduced AQP4 expression is reported in posterior cortical regions and the ventral cortex [136], although other studies demonstrate the upregulation of global AQP4 expression even if depolarized [142,143]. Deficiency of AQP4 expression and loss of astrocytic polarization were directly linked to impaired solute clearance leading to phosphorylated tau (P-tau) accumulation in both the contralateral and ipsilateral hemispheres, exacerbated neurodegeneration, and cognitive deficits [144]. On the other hand, small interfering RNA-mediated inhibition of *AQP4* significantly reduced brain edema in the acute phase post-TBI (6 to 12 h) [145]. Temporal patterns of AQP4 expression post-TBI provide further insights into this controversy. Levels of AQP4 seem to peak early during the acute phase (12 and 72 h post-TBI) and decline later (by day 15) [142]. These changes are closely associated with the volume of brain water content and the severity of the edema. Notably, although AQP4 has an important role in edema formation, its function in the long term is compensatory, assisting in resolution once the BBB stability has been restored [143]. However, other studies have noted the emergence of waste clearance dysfunction around 28 days post-injury in a blast mild TBI model [141].

Across studies of mild TBI (mTBI), subacute TBI, and chronic TBI, glymphatic function was assessed using the DTI-ALPS index and the estimation of ePVS burden. A consistent impairment of waste clearance was reported [146,147,148,149]. Reduced DTI-ALPS index correlated with decline in cognitive performance [147], including verbal memory, attention, and executive function [146]. Notably, the DTI-ALPS index may have a prognostic utility for identifying chronic cognitive impairment following TBI [29], while the cognitive decline was not related to factors reflecting clinical burden at the baseline [147]. Collectively, these findings provide cues to consider a link between the DTI-ALPS index and cognitive decline that relies predominantly on clearance mechanisms.

Alongside reduced DTI-ALPS values, patients with mTBI had profound global white-matter damage [146]. Global white-matter damage partially mediated the association between the DTI-ALPS index and cognition. Furthermore, the DTI-ALPS index was negatively correlated with plasma neurofilament light (NfL) expression, linking reduced DTI-ALPS to the severity of axonal damage biology [149]. These observations suggest that impaired glymphatic flow is present in more severe or diffuse injury phenotypes [146,148,149], pointing towards a relationship between glymphatic dysfunction and diffuse white-matter integrity to explain cognitive deficits after mTBI [146] (Supplementary Table S1).

Conversely, higher DTI-ALPS values have been reported in mTBI cohorts, including MRI-negative cases, while longitudinal increases in DTI-ALPS have also been observed [150]. A gradual increase of the DTI-ALPS index from the acute (14 days post-injury) to the chronic phase (6–12 months post-injury) was noticed [29] (Supplementary Table S1). These findings indicate time-dependent changes in perivascular diffusivity after injury. Whether these changes represent compensatory mechanisms, tissue structure alterations, or other recovery-related processes remains uncertain. A compensatory mechanism can be hypothesized based on findings reporting that improvements in sleep quality are associated with increased DTI-ALPS values [29]. Additionally, these dynamic fluctuations of glymphatic activity in TBI render the DTI-ALPS index a very useful marker in interpreting the correlation between time- and context-dependent shifts in perivascular diffusivity and clinical outcomes.

Collectively, these studies reinforce the growing understanding that glymphatic dysfunction and AQP4 in particular play a significant role in TBI pathophysiology. Chronic impairment of glymphatic flow post-TBI may render the post-traumatic brain prone to the accumulation of neurotoxic proteins that lead to neurodegeneration [144]. This is supported by the restoration of the glymphatic function exerted by some of the therapeutic strategies applied in experimental models such as omega-3 polyunsaturated fatty acids [151], adrenergic blockers [139], and GLP-1R agonists [152], which are associated with BBB preservation, improvement of neurological outcomes, and reduced accumulation of inflammatory components.

## 4. Inflammaging

Enhanced depolarization of AQP4 as well as altered CSF dynamics point towards a deregulation of waste clearance mechanisms during aging. Increased expression of AQP4 with advancing age has been detected in humans and mice [153,154]. AQP4 staining in old, non-demented individuals shows a distinct diffuse parenchymal pattern compared to the perivascular polarization observed in younger individuals. Enlarged PVS and increased water pool is also observed in aging brains [155], pointing towards a reduction in the CSF turnover [10] and impediment of CSF flow [156]. Besides impaired glymphatic flow, draining to meningeal lymphatic vessels was also found to be related to aging in patients undergoing 3-dimensional T1-weighted imaging following intrathecal injection of gadolinium [157]. These observations suggest a holistic glymphatic flow stagnation in aged individuals and related experimental models.

Although limited, evidence points towards an association between glymphatic impairment and aging that leads to neuroinflammatory conditions. The presence of excessive immune cells in PVS due to chronic low-grade inflammation [158], as well as the reduced arterial pulsatility [12], could explain mechanistically the reduced CSF flow. Immune-mediated loss of meningeal drainage capacity has been observed in old mice [159]. Moreover, a substantial decline in the fluid exchange between CSF and the brain parenchyma in mice of advanced age (18 months old) compared to younger ones (2–3 months old) was accompanied by reduced clearance of injected Aβ and loss of perivascular polarization of AQP4 [8]. Notably, AQP4 was found to be localized in the areas that affect the recirculation of CSF into and through the brain interstitium, while its expression was elevated in areas where the penetration of CSF tracer was reduced, such as the pial surface. Depolarization of AQP4 is also associated with age-related cognitive decline and increased Aβ burden [31], suggesting AQP4-dependent cognitive deterioration. Although this is preliminary evidence, a feedback loop could be suggested: chronic inflammation leads to the accumulation of immune mediators within CNS, hampering the glymphatic flow and subsequently fostering protein aggregation, resulting in enhanced neuroinflammation and further obstruction of glymphatic flow. This theoretical mechanism could thus contribute to the onset of neurocognitive and neurodegenerative conditions.

## 5. Gut–Brain Axis

Recent evidence points to a possible interplay between the gut–brain inflammatory axis and the deregulation of waste clearance within CNS. Indirect effects of gut dysbiosis may have an impact on glymphatic function via several mechanisms [160]. In particular, gut dysbiosis may result in the accumulation of pro-inflammatory mediators in the periphery as well as the activation of peripheral immune cells. Following their migration within CNS, signaling effects in local glial cells may enhance the neuroinflammatory burden with the potential to halt the glymphatic flow [5]. Besides blood circulation, additional routes, such as the vagus nerve, allow for direct communication between the gut and the brain, thus enabling the local build-up of inflammation that may result in the aggregation of neurotoxic peptides via a feedback loop [161]. Moreover, gut microbiota regulate the release of neurotransmitters and neuroactive hormones that may perturb astrocytic functions, rendering them incapable of performing their role in the regulation of glymphatic flow and waste clearance [162]. Gut dysbiosis may also lead to dysregulation of BBB integrity through the release of short-chain fatty acids [163]. Given that BBB is suspected to serve as a complementary mechanism to the glymphatic system [164], its dysregulation can compromise the waste removal machinery. The composition of gut microbiota is further associated with the regulation of circadian rhythms, which are directly related to the activity of the glymphatic system. Indeed, microbiota dysbiosis may generate potent activators of aryl hydrocarbon receptors, which are able to disrupt circadian rhythms via sleep fragmentation [165] and subsequently impair glymphatic flow [166].

We can further speculate on the possible impact of gut dysbiosis in the aggregation of neurotoxic proteins within the CNS, focusing on studies that employ a gut-targeted modification of microflora. Gut dysbiosis induced by administration of periodontal pathogen in mice has been shown to be associated with increased Aβ accumulation alongside systemic and focal CNS inflammation and reactive astrogliosis [167]. Additionally, dextran-sulfate-sodium-induced colitis in aged mice led to extended gut inflammation that was accompanied by increased astrogliosis, impaired glymphatic clearance, depolarization of AQP4, and increased Aβ accumulation [168]. In this specific experimental model, glymphatic function was restored and neuroinflammation was mitigated following pharmacological inhibition of the NLRP3 inflammasome, suggesting an interconnectedness of gut-induced neuroinflammation and waste clearance impairment.

Existing evidence points towards a hypothetic mechanistic interplay between secreted metabolites by the gut and CNS homeostasis, with waste clearance within the CNS being one of the domains affected. However, more research is warranted to confirm that this existing interaction may lead to the deregulation of waste clearance processes from the brain.

## 6. Regulation of Glymphatic Function

### 6.1. Sleep

Injected trackers in the cisterna magna or the choroid plexus have yielded initial findings supporting that clearance of solutes from the brain is accelerated in either natural sleeping or anesthetized mice compared to awake mice, while sleep deprivation led to reduced CSF influx [169,170]. Increased CSF influx is proposed to occur due to the expansion of ISF volume, thus facilitating the fluid interchange [171]. Furthermore, sleep fragmentation induced via tilting platforms in 5X FAD mice resulted in compromised short-term and spatial memory accompanied by increased accumulation of Aβ and astrogliosis [172]. The expression of AQP4 was deregulated both in wild-type and 5X FAD mice following sleep deprivation, suggesting that defects in waste clearance may be associated with cognitive deficits.

Using the DTI-ALPS index, Saito and colleagues have recently shown that perivascular diffusivity was significantly lower in a group of patients with sleep disruption compared to individuals with normal sleep [173]. Furthermore, impaired clearance has been demonstrated in patients with obstructive sleep apnea [174]. Altered glymphatic flow has been further correlated with chronic sleep disturbance in patients with iNPH compared to non-demented individuals [175]. Notably, sleep disturbances have been associated with behavioral disturbances in PD patients [176], and tau accumulation and faster cognitive deterioration in AD patients [177]. In contrast, Miao et al. found in mice that fluorescent tracer clearance was reduced during both sleep and anesthesia compared with wakefulness, while tracer movement within the parenchyma was largely independent of vigilance state, challenging the theory of toxin clearance during sleep [178]. Thus, sleep-associated changes in tracer influx or distribution may not necessarily be interpreted as evidence of increased clearance. Possibly, methodological differences including tracer administration, imaging methodology, the stage of sleep where the assessment was performed, and the specific outcome used to define solute clearance may contribute to apparently conflicting findings, warranting increased caution in interpretation [179].

Vasomotion has recently emerged as a potentially important link between sleep architecture and brain fluid transport [180]. Chronic sleep fragmentation in mice suppresses slow vasomotion and reduces interstitial clearance despite relatively preserved cardiac-driven pulsations, suggesting that low-frequency vascular oscillations may contribute independently to glymphatic-related transport [181]. Moreover, infraslow norepinephrine oscillations during non-rapid eye movement (NREM) sleep have been reported to coordinate cerebral vasomotion and reciprocal CSF dynamics; experimentally induced arterial oscillations increased CSF influx, whereas zolpidem attenuated norepinephrine oscillations and glymphatic transport [180]. Emerging human evidence further indicates that sleep pressure and slow-wave-rich sleep differentially affect low-frequency vasomotor, respiratory, and cardiac brain pulsations, supporting the presence of vascular mechanisms capable of increasing CSF flow to the brain [182]. Nevertheless, whether these sleep-associated oscillations translate into greater clearance remains debated, due to other communications reporting reduced brain tracer clearance during natural sleep and anesthesia relative to wakefulness [178]. These conflicting observations emphasize that CSF influx, perivascular mixing, and net tissue clearance may represent distinct processes and may potentially be regulated by different mechanisms during sleep. Overall, additional research is warranted to unravel the pathways involved in solute clearance during sleep.

### 6.2. Anesthesia

Anesthetic state seems to influence glymphatic function through its effects on neural oscillations, vascular pulsatility, and adrenergic tone. Rodents anesthetized with ketamine/xylazine show higher CSF tracer influx into the brain compared to mice under isoflurane alone or other agents [183]. It is not clear, though, whether elevated CSF influx depends on increased CSF production, since contradictory results have been published [184,185]. Enhanced influx is coupled to the induction of slow-wave electroencephalogram activity, which promotes low noradrenergic signaling and synchronized neuronal activity that widens perivascular spaces, thereby facilitating CSF–ISF exchange. In contrast, anesthetics such as isoflurane, which generate lighter sedation with higher heart rates and less delta power, do not have a substantial impact on glymphatic transport. Thus, deeper slow-wave oscillatory activity under certain anesthetics appears to facilitate perivascular CSF entry into the parenchyma.

Structural changes following anesthesia also confer altered glymphatic function. Prolonged or high-dose isoflurane disrupts AQP4 localization, impairing solute efflux and leading to accumulation of inflammatory proteins and Aβ, phenomena associated with postoperative cognitive decline [186]. Restoration of AQP4 polarization could partially preserve waste clearance and neurocognitive performance. Further, co-administration of dexmedetomidine, an α2-adrenergic agonist that reduces locus coeruleus activity, with low-dose isoflurane enhances glymphatic transport compared to isoflurane alone, pointing towards a dependency on the noradrenergic tone and vascular dynamics [187].

These findings underscore a two-sided effect of anesthesia: when high-dose isoflurane is administered or during prolonged exposure, glymphatic function may be stalled, while the usage of anesthetics that promote slow-wave neuronal states leads to maintenance of vascular pulsatility and AQP4 polarization. Reduced adrenergic signaling results in relaxed perivascular spaces and an expansion of interstitial spaces [169] that subsequently lowers vascular resistance, thus promoting clearance [187].

### 6.3. Sensory Stimulation

High-intensity visual stimulation using a flickering checkerboard has been shown to induce macroscopic CSF flow in the fourth ventricle in awake humans [188]. Findings further supported that neural activity may modulate CSF flow via neurovascular coupling. A more recent report supports these observations by extending these observations to age-related neurodegenerative conditions of the visual system, such as glaucoma [189]. Using task-based fMRI, Bang and colleagues demonstrated that the characteristic stimulus-locked CSF patterns and their coupling with visual-cortex blood-oxygenation-level-dependent response diminish with increasing disease severity, suggesting that disease-related impairment of neuronal and neurovascular responses may disrupt sensory-driven CSF dynamics. Further, preclinical evidence from the murine 5XFAD experimental model supports a potential relationship between sensory stimulation and glymphatic-related clearance [190]. Combined auditory and visual stimulation at 40 Hz increased cortical CSF influx and interstitial fluid efflux, enhanced AQP4 polarization along the astrocytic endfeet, and meningeal lymphatic dilation. Importantly, amyloid removal was enhanced following multisensory stimulation, suggesting that arterial pulsatility is a key phenomenon for effective removal. Collectively, these findings suggest that sensory-stimulation-dependent neurovascular dynamics may represent an additional regulator of CSF movement and potentially glymphatic-related transport. However, evidence that sensory stimulation produces clinically meaningful enhancement of glymphatic clearance in humans remains lacking. Therefore, more research is warranted to confirm these observations in the clinical setting.

### 6.4. Body Posture

Changes in posture may have an impact on CSF circulation [191]. Furthermore, CSF pressure and volume are influenced by posture due to gravity [192]. This is crucial since elevated intracranial pressure has been implicated in several neurodegenerative conditions [193]. Additionally, postoperative cognitive impairment has been associated with the acquired posture during surgery [194]. These findings suggest an interplay between body posture and altered CSF dynamics that may have an impact on brain functions.

Using MRI and fluorescence tracers in rodents, researchers have shown that Aβ clearance is significantly superior in the lateral position compared to the supine or prone positions, suggesting the existence of an evolved mechanism to sustain brain homeostatic glymphatic function [195]. Similarly, in human subjects, the CSF exchange seems to be enhanced in the supine compared to the upright position, providing benefits for brain waste clearance processes [196]. The supine position seems to be ideal for the glymphatic process, possibly by increasing intracranial pressure, which subsequently allows for more CSF movements and higher driving pressure [197]. More insights concerning the relationship between body posture and glymphatic function are warranted to elucidate the mechanisms involved.

### 6.5. Alcohol

Alcohol intake has been shown to exert dose-dependent and temporally distinct effects on glymphatic function. Acute and chronic exposure to high levels of ethanol (>1.5 g/kg) significantly suppresses glymphatic transport, leading to impaired CSF influx and reduced clearance of waste metabolites [198,199]. Suppression is associated with astrogliosis, AQP4 depolarization, and reduced cerebrovascular pulsatility, thus contributing to compromised Aβ clearance [199]. By contrast, low-dose ethanol exposure (0.5 g/kg) appears to enhance glymphatic activity, which is accompanied by reduced GFAP expression and stabilization of AQP4 polarization [198]. Furthermore, in humans, acute moderate alcohol administration induces reversible ventricular enlargement, possibly reflecting a shift in CSF dynamics and water volume, suggesting a glymphatic system malfunction [200].

Mechanistically, ethanol-induced vasodilation following low exposure to alcohol may enhance vascular reactivity, thereby promoting perivascular clearance of larger solutes and proteins [201]. Higher volumes of alcohol may have a detrimental effect, inducing astrocytic amyloid generation and enhanced astrogliosis [202] as well as in vivo depolarization of AQP4 [203]. Increased astrogliosis was also present in human postmortem studies of patients with alcohol use disorder [204].

Collectively, these observations suggest that alcohol modulates glymphatic clearance in a dose-dependent manner. Low-dose ethanol may transiently enhance waste clearance through increased vascular pulsatility, whereas chronic or high-dose exposure results in glymphatic flow stagnation. However, it should be noted that the reported enhancement of glymphatic activity following low-dose ethanol derives predominantly from preclinical evidence and should be interpreted with caution.

## 7. Conclusions

The evidence synthesized in this review provides available data that support the contention that glymphatic system dysfunction may constitute a common clinically relevant feature across neurodegenerative and neuroinflammatory conditions. However, it is not clear whether glymphatic dysfunction is a mechanism preceding neurodegeneration or is an epiphenomenon of underlying pathogenesis. Dysfunction appears as a vulnerability that links aging, inflammation, and neurodegeneration. More importantly, glymphatic dysfunction seems to be detectable in prodromal and early-stage disease phenotypes, implying that a waste clearance deterioration may precede initial stages of disease pathogenesis. These findings converge on the notion that glymphatic dysfunction may be among the drivers of disease onset. Therefore, we cannot make concrete conclusions concerning the potential role of the glymphatic system, while we highlight the need for future experiments that will decipher this experimental void in our knowledge.

Altered brain homeostasis due to impaired glymphatic flow appears potentially to lead to CNS function deficits, including cognitive decline among others. Cognitive deficits dominate findings of human imaging studies, indicating sensitivity of brain regions such as the hippocampus and temporal cortex due to accumulated waste [205]. Furthermore, the recurrence of glymphatic-related abnormalities across neurological disorders raises the possibility of a shared clearance-related vulnerability rather than a disease-specific process. With respect to all domains that need to be validated, preclinical evidence may point to inefficient waste clearance as a mechanism contributing to neurodegeneration and neuroinflammation, although available human evidence does not establish whether these alterations are causal, contributory, compensatory, or secondary to the underlying pathology. The variable interplay of the glymphatic system with other waste clearance pathways should be explored further to gain insights regarding different pathways of neurodegeneration [206].

Another limitation regarding pre-clinical models lies in the translation of experimental findings to humans, due to species differences; for example, humans exhibit higher anatomical complexity compared to rodents [207], while the latter present higher AQP4 perivascular polarization compared to the former [208]. However, various evidence from human imaging studies across AD, PD, ALS, MS, FTD, stroke, and TBI consistently demonstrates altered glymphatic function. Therefore, we believe that this issue does not fall within the limitations of this synthesis. In most human imaging studies, glymphatic function has been assessed with the DTI-ALPS index, which serves as a proxy. Low DTI-ALPS indices are often associated with increased perivascular space burden and choroid plexus abnormalities. These observations support a model in which impaired clearance of aggregation-prone proteins, inflammatory mediators, and metabolic byproducts contributes to a neuroinflammatory cascade that potentially leads to neuronal dysfunction. It should be noted that the DTI-ALPS is a practical, non-invasive surrogate marker of glymphatic function; however, limitations concerning its application exist. The method assumes that diffusivity along PVS reflects glymphatic activity. Yet, diffusion anisotropy may also be influenced by other factors including white-matter microstructure, axonal degeneration, edema, and small vessel disease, potentially confounding interpretation of outcomes [26]. Moreover, the DTI-ALPS index is a snapshot of water diffusivity, and not representative of dynamic flow. Therefore, its application can be influenced by diffusion as well as convective flow, which has given rise to a lively debate concerning glymphatic biology [209]. Lack of established outputs as well as variability of monitoring methods may also reduce variability until a consensus is reached. Thus, although DTI-ALPS is a useful clinical tool, its outcomes should be interpreted with caution and in combination with other endpoints to properly assess glymphatic function in clinical routine. Future experiments should be focused on the functional improvement of DTI-ALPS as well as the development of robust and reliable diagnostic markers for the assessment of glymphatic capacity in humans.

Glymphatic function seems to be strongly dependent on the function of AQP4. Through our synthesis, the role of AQP4 is framed as a central facilitator of glymphatic transport due to its polarized expression at astrocytic perivascular endfeet. Initial experiments using *Aqp4* knockout mice have supported this mechanistic link [1]. However, subsequent studies employing models of *Aqp4* deletion have questioned its role in the magnitude of parenchymal tracer spread [210]. Additionally, others have drawn attention to technical issues such as the type of anesthesia used, and the timing as well as the tracer delivery route may influence our understanding of the role of AQP4 [211]. Thus, while AQP4 likely enhances perivascular CSF–ISF exchange by increasing astrocytic water permeability, whether it is essential for large-scale convective glymphatic flow or instead modulates local mixing and dispersion remains unresolved.

Age-associated structural changes in vascular integrity, alterations in astrocyte physiology with prominent AQP4 depolarization, and systemic inflammation caused by gut dysbiosis may result in gradually accumulated inflammatory burden and loss of BBB integrity. Glymphatic dysfunction driven by aging, alongside these background conditions, may render the CNS environment vulnerable to neurodegeneration onset. This bidirectional interaction suggests that inflammaging and glymphatic failure work in parallel, providing a theoretical mechanism for explanation of the strong age-dependent risk of progression of neurodegeneration. Within this model, glymphatic dysfunction may result in retention of immunogenic components, thus amplifying inflammatory signaling, thereby accelerating pathogenic cascades. In the opposite direction, accumulated inflammatory burden may result in glymphatic efflux stagnation, further enhancing the presence of toxic metabolic byproducts and aggregation-prone proteins that cause neuronal damage.

## 8. Future Directions

Collectively, current imaging evidence positions glymphatic assessment as a promising research biomarker, rather than an established clinical tool [2]. Development of biomarkers based on non-invasive MRI-based methods, particularly DTI-ALPS, offer practical advantages for longitudinal investigation, but clinical translation will require harmonized acquisition and processing protocols, as well as the establishment of a consensus among physicians concerning normative reference ranges, validated thresholds, reproducibility, and increased diagnostic and prognostic value beyond established biomarkers. Multimodal assessment coupling diffusion imaging with structural PVS measures, choroid plexus imaging, vascular and sleep metrics, as well as molecular biomarkers may enable earlier detection of brain vulnerabilities and risk-stratification-based prognosis. Eventually, harnessing the modalities of the glymphatic system could open the path for the development and repurposing of cross-disease therapeutic strategies, given its involvement in multiple neurodegenerative pathways.

## Figures and Tables

**Figure 1:**
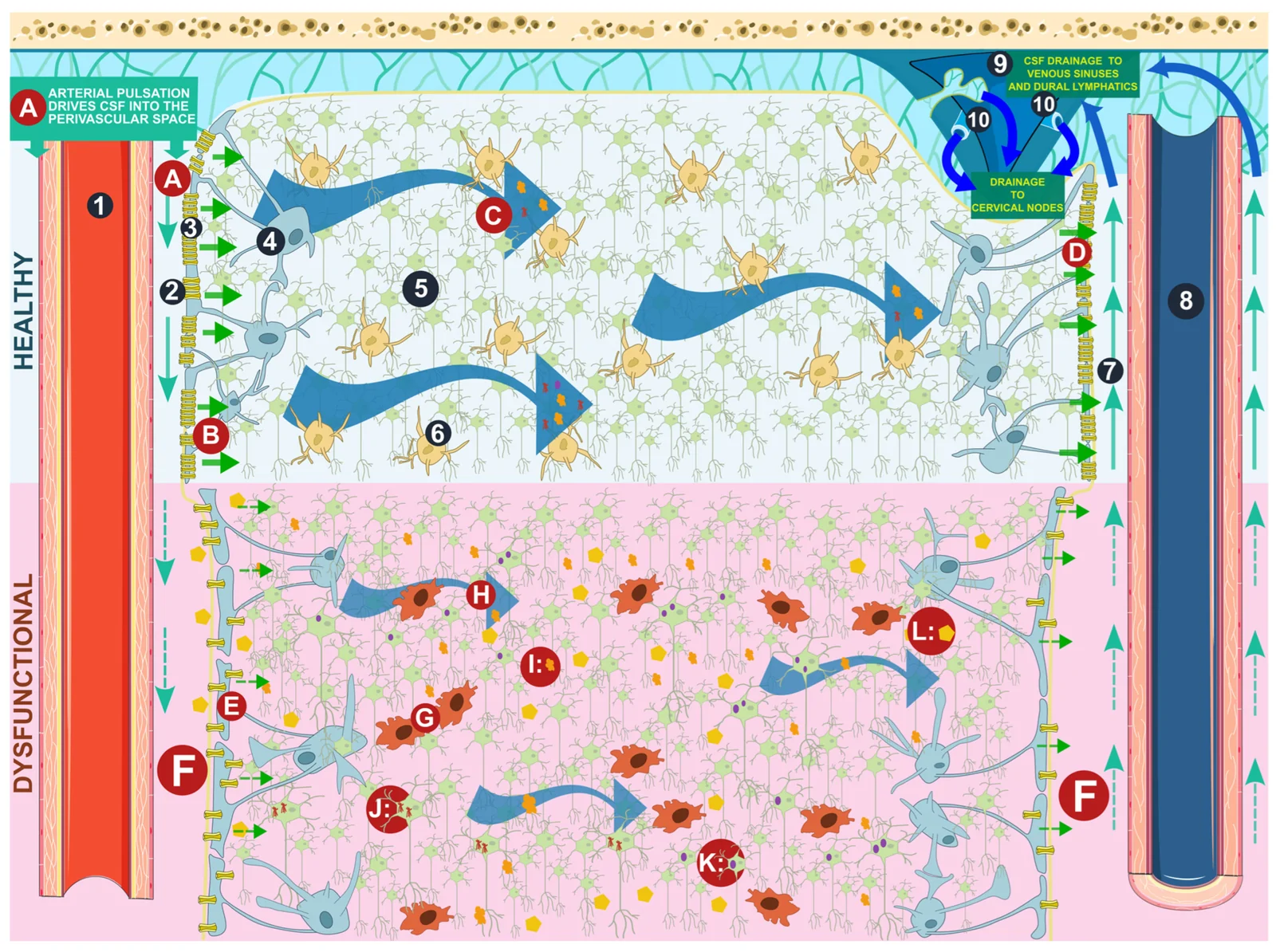
Glymphatic system physiology and dysfunction as a convergent mechanism across CNS disorders. Cross-sectional schematic of cerebral parenchyma, comparing healthy (**upper row**) and dysfunctional (**lower row**) glymphatic system function. The glymphatic system is a brain-wide perivascular network that facilitates cerebrospinal-fluid–interstitial-fluid exchange and clearance of metabolic waste. CSF enters (A) the brain along periarterial spaces (2) of penetrating arterioles (1), driven by arterial pulsatility and pressure gradients, and crosses into the interstitium through aquaporin-4 (AQP4) water channels (3) expressed on the endfeet of astrocytes (4). The endfeet ensheathe the cerebral vasculature and form the outer boundary of the perivascular spaces, with proper polarization (B) of AQP4 channels at the endfeet being crucial for glymphatic function. A convective CSF and ISF bulk flow (C) arises through the brain parenchyma, populated by neurons (5) and surveillant ramified microglia (6). The mixed CSF–ISF then exits (D) the brain along perivenous spaces (7) of venules (8), draining toward the venous sinuses (9) and dural lymphatic vessels (10), ultimately towards the cervical lymph nodes (not depicted). Glymphatic function contributes to the clearance of neurotoxic byproducts, including amyloid-β (Aβ), tau, and α-synuclein. In the dysfunctional state (lower row), loss of perivascular localization of AQP4 (depolarization, E) impairs CSF–ISF exchange and waste clearance, while the perivascular spaces become enlarged (F). Microglia cells transition from ramified to amoeboid morphology, indicating activation (G). Decreased glymphatic flow (H) may result in accumulation of Aβ plaques (I), tau neurofibrillary tangles (J), α-synuclein aggregates (K), and pro-inflammatory cytokines (L), contributing to neuroinflammation and neuronal dysfunction. Images provided by Servier Medical Art (https://smart.servier.com/), licensed under CC BY 4.0 (https://creativecommons.org/licenses/by/4.0/) (accessed data 30 July 2026).

## Data Availability

No new data were created or analyzed in this study. Data sharing is not applicable to this article.

## Supplementary Materials

The following supporting information can be downloaded at: https://www.mdpi.com/article/10.3390/biomedicines14102178/s1, Table S1: Summary of human studies assessing glymphatic-related imaging markers across neurological disorders.

## Abbreviations

The following abbreviations are used in this manuscript:

Aβ	Amyloid-beta
AD	Alzheimer’s disease
ALS	Amyotrophic lateral sclerosis
ALPS	Analysis along the perivascular space
AQP4	Aquaporin-4
APP/PS1	Amyloid precursor protein/Presenilin-1
BBB	Blood–brain barrier
bvFTD	Behavioral variant frontotemporal dementia
CNS	Central nervous system
CSF	Cerebrospinal fluid
CSVD	Cerebral small vessel disease
DTI	Diffusion tensor imaging
DTI-ALPS	Diffusion tensor imaging along the perivascular space
EAE	Experimental autoimmune encephalomyelitis
ePVS	Enlarged perivascular spaces
FTD	Frontotemporal dementia
GFAP	Glial fibrillary acidic protein
GLP-1R	Glucagon-like peptide-1 receptor
ICH	Intracerebral hemorrhage
iNPH	Idiopathic normal pressure hydrocephalus
ISF	Interstitial fluid
MCAO	Middle cerebral artery occlusion
MCI	Mild cognitive impairment
MMSE	Mini-Mental State Examination
MoCA	Montreal Cognitive Assessment
MOG	Myelin oligodendrocyte glycoprotein
MRI	Magnetic resonance imaging
MS	Multiple sclerosis
NfL	Neurofilament light
NLRP3	NOD-, LRR- and pyrin domain-containing protein 3
PD	Parkinson’s disease
PFFs	Preformed fibrils
PVS	Perivascular spaces
SAH	Subarachnoid hemorrhage
SCD	Subjective cognitive decline
SNCA	Alpha-synuclein (gene/protein)
SOD1	Superoxide dismutase 1
TBI	Traumatic brain injury
TDP-43	TAR DNA-binding protein 43
UPDRS	Unified Parkinson’s Disease Rating Scale
VCID	Vascular cognitive impairment and dementia
VTN	Ventral thalamic nucleus
5XFAD	Five familial Alzheimer’s disease transgenic mouse model
