# Supplementary material for: Glymphatic Dysfunction Across Neurological Disorders: Links to Neurodegeneration, Inflammaging, Gut–Brain Signaling, and Physiological Modulators
Source: Biomedicines. 2026 Sep 25;14(10):2178. doi: 10.3390/biomedicines14102178 (PMC13701315; doi:10.3390/biomedicines14102178)
Supplement: Supplementary file 1 [file biomedicines-14-02178-s001.zip › biomedicines-4504843-supplementary.pdf]

Supplementary Table s1: Summary of human studies assessing glymphatic-related imaging markers across neurological disorders

Condition	Study	Participants	Method	Main findings
Alzheimer's disease and prodromal stages	Bao et al, 2025[1]	N=253 cognitively normal<br>N=266 MCI	DTI-ALPS	Higher ALPS index was associated with a lower risk of MCI and with an approximately 3.5-year delay in progression from MCI to AD. This association may be partially mediated by Aβ accumulation in the choroid plexus, cortical tau burden, and executive function.
	Kamagata et al, 2022[2]	N=36 AD<br>N=44 MCI<br>N=31 healthy controls	PVS volume fraction, fractional volume of free water in white matter (FW-WM), and DTI-ALPS	AD showed lower ALPS and greater PVS burden/free water than HC. Lower ALPS was associated with lower CSF Aβ42, reduced FDG uptake and poorer cognition.
	Li Y et al, 2024[3]	N=654 from Sino Longitudinal Study on Cognitive Decline<br>N=650 from the Alzheimer's Disease Neuroimaging Initiative	DTI-ALPS	ALPS abnormalities were detectable already in subjective cognitive decline while lower ALPS was associated with amyloid burden and cognitive progression across the AD continuum.
	Schirge et al, 2025[4]	ActiGlia<br>N=16 AD<br>N=18 controls<br>DELCODE<br>N=54 AD<br>N=67 controls<br>ADNI<br>N=43 AD<br>N=49 controls	DTI-ALPS<br>FLAIR MRI for WMH	AD showed reproducibly lower ALPS across independent cohorts; lower ALPS was associated with amyloid-related and cognitive/clinical measures and WMH burden.
	Huang et al, 2024[5]	N=47 AD dementia<br>N=137 MCI<br>N=235 controls	DTI-ALPS	Lower ALPS predicted faster amyloid accumulation, brain atrophy, clinical progression and cognitive decline; amyloid and atrophy mediated part of the association with cognition.

	Steward et al, 2021[6]	N=16 AD<br>N=10 MCI<br>N=10 controls	DTI-ALPS	Right-sided ALPS differed between cognitively normal participants and AD/MCI and correlated with MMSE and ADAS-Cog11.
	Zhang et al, 2024[7]	N=15 AD<br>N=15 MCI<br>N=26 controls	DTI-ALPS, PVS volume	AD dementia showed lower ALPS and greater basal-ganglia PVS burden; lower ALPS correlated with PVS enlargement and poorer cognitive performance.
Parkinson's disease	Ren et al, 2025[8]	N=139 PD<br>N=62 controls	Longitudinal DTI-ALPS	ALPS was lower in PD; lower baseline ALPS was associated with more rapid motor progression during longitudinal follow-up.
	Zhao et al, 2025[9]	N=51 PD<br>N=30 controls	3T MRI/DTI-ALPS, structural MRI	PD showed lower ALPS. Lower ALPS was associated with cognitive impairment and cortical degeneration; cortical atrophy partly mediated the association with cognition.
	Yao et al, 2024[10]	N=25 PD<br>N=30 controls	^{18}F-DOPA PET-MRI scanning<br>DTI-ALPS<br>CPV and EPS volumes	PD showed lower ALPS and larger CPV/ePVS volumes. ALPS correlated negatively with UPDRS-III and positively with dopaminergic PET uptake.
	Cai et al, 2023[11]	N=93 PD<br>N=42 controls	DTI-ALPS	PD showed lower ALPS; lower values were associated with older age, older age at onset and poorer sleep, with an association with motor severity particularly in older patients.
	Bae et al, 2023[12]	N=54 PD<br>N=54 controls	DTI with concurrent ^{123}I-2β-carbomethoxy-3β-(4-iodophenyl)-N-(3-fluoropropyl)-nortropane (^{123}I-FP-CIT) SPECT	Lower ALPS in PD correlated with worse UPDRS-III, MMSE and MoCA scores, but not with striatal dopamine-transporter uptake.
	Si et al, 2022[13]	N=168 PD (among them N=50 longitudinally reexamined)<br>N=119 possible iRBD<br>N=129 controls	DTI-ALPS	ALPS was lower in PD and possible iRBD than HC and was inversely related to disease severity; longitudinally, lower ALPS was associated with cognitive decline in PD.
	Zhou et al, 2024[14]	N=175 PD; N=103 followed for 5 years	DTI-ALPS	Lower ALPS independently predicted clinically important milestones, including recurrent falls, wheelchair dependence and dementia.

Frontotemporal dementia	Premi et al, 2024[15]	N=291 symptomatic or presymptomatic FTD<br>N=83 non-carriers	DTI-ALPS; longitudinal assessment in N=180	Symptomatic mutation carriers had lower ALPS than presymptomatic carriers and older non-carriers. Lower ALPS was associated with greater disease severity and faster subsequent progression.
	Jiang et al, 2023[16]	N=74 bvFTD<br>N=67 controls	CP volume, DTI-ALPS along ^{18}F-FDG-PET	bvFTD showed regional glymphatic-related abnormalities, particularly anteriorly/mid-brain, including altered ALPS, weaker anterior BOLD-CSF coupling and increased CP volume; measures were related to cognition and disease severity.
	Xiao et al, 2024[17]	N=122 FTD; N=56 bvFTD, N=30 svPPA, N=36 nfvPPA	DTI-ALPS, WMH, cortical morphology	DTI-ALPS alterations differed according to FTD subtype and were related to cortical thickness, WMH and cognitive abnormalities.
Idiopathic normal-pressure hydrocephalus	Ringstad et al, 2017[18]	N=15 iNPH<br>N=8 reference subjects	Serial T1 MRI after intrathecal contrast using intrathecal gadobutrol clearance	iNPH showed ventricular reflux, delayed tracer enhancement and reduced clearance, with persistent parenchymal tracer enrichment.
	Ringstad et al, 2018[19]	N=9 iNPH<br>N=8 reference subjects	Serial T1 MRI after intrathecal contrast using intrathecal gadobutrol clearance	Demonstrated brain-wide CSF-tracer penetration; iNPH showed significantly greater tracer retention at 24 h in multiple regions, consistent with delayed clearance.
	Eide et al, 2019[20]	N=30 iNPH<br>N=8 reference subjects	gMRI acquisitions were obtained over a 24-48 h time span using intrathecal gadobutrol clearance	Delayed tracer clearance from CSF, entorhinal cortex and adjacent white matter in iNPH, accompanying structural vulnerability of memory-related regions.
Amyotrophic lateral sclerosis	Huang et al, 2025[21]	N=30 ALS<br>N=38 controls	DTI-ALPS, resting-state fMRI	ALS showed lower ALPS and altered diffusion, accompanied by reduced sensorimotor ALFF. ALPS-related measures correlated with neural activity and clinical severity/duration.
	Choi et al, 2025[22]	N=51 ALS<br>N=51 controls	DTI-ALPS, CPV/total intracranial volume	ALS showed lower ALPS and larger CP fraction; ALPS and CP fraction were inversely related. Both were age-associated but not significantly associated with disability/progression.
	Baek et al, 2025[23]	N=35 ALS<br>N=35 PD<br>N=13 controls	DTI-ALPS	Right-sided ALPS was lower in ALS than normal controls; no significant difference between ALS and PD patients. ALPS declined with age but was not related to ALS clinical severity.

	Liu et al, 2024[24]	N=56 early-stage ALS<br>N=32 controls	DTI-ALPS	Early ALS showed lower ALPS; lower ALPS was associated with clinical disability and sleep abnormalities, particularly sleep efficiency and periodic limb movements.
	Sharkey et al, 2024[25]	N=18 ALS<br>N=5 PLS<br>N=22 controls	Longitudinal DTI-ALPS at baseline, 4 and 8 months	ALS showed persistently lower ALPS than both PLS and HC across all three time points, indicating persistence of the imaging abnormality over 8 months.
Multiple Sclerosis	Carotenuto et al, 2022[26]	N=71 MS; N=49 RRMS, N=22 PMS<br>N=32 controls	Multimodal MRI, DTI-ALPS	ALPS was lower in MS than HC and more severely reduced in progressive MS. Lower ALPS was associated with greater disability, lesion burden, grey-matter atrophy and diffuse WM injury.
	Preziosa et al, 2025[27]	N=146 MS; N=103 RRMS, N=43 PMS<br>N=72 controls	DTI-ALPS, CP volume	MS showed lower ALPS and cortical volumes and larger CP volume. ALPS was associated with lesion burden and lower brain volumes and partially mediated the association between CP enlargement and brain damage.
	Bayoumi et al, 2024[28]	N=118 MS; N=57 lower disability, N=61 higher disability	DTI-ALPS, brain parenchymal fraction	Higher-disability patients had lower ALPS; ALPS correlated inversely with EDSS and disease duration and was related to brain parenchymal fraction and lesion load.
Autoimmune encephalitis	Zhang et al, 2025[29]	N=85 AE<br>N=30 controls	Longitudinal DTI-ALPS	Baseline ALPS was lower in autoimmune encephalitis and correlated with MMSE/MoCA. ALPS increased during follow-up, while low baseline ALPS predicted greater risk of cognitive impairment at 12 months.
Stroke and cerebral small-vessel disease	Lin et al, 2025[30]	N=82 cerebral infarction; N=40 good prognosis group, N=42 poor prognosis group	DTI-ALPS	Poor-prognosis patients had lower admission ALPS; ALPS indices were associated with neurological/functional outcome and were independent predictors of poor prognosis.
	Zhang et al, 2022[31]	N=20 sICH<br>N=31 controls	DTI-ALPS, susceptibility-weighted imaging scanning	Lesion-side ALPS was lower than both the contralateral hemisphere and HC; lesion-side ALPS correlated with disease duration.
	Zhang et al, 2025[32]	N=51 ischemic stroke<br>N=47 nonvascular cognitive impairment<br>N=48 controls	Longitudinal DTI-ALPS between pre- and post-rehabilitation. CP volume, PVS volume	Stroke patients had lower bilateral ALPS, particularly ipsilesionally; infarct-side ALPS increased after rehabilitation and baseline ALPS was associated with 6-month PSCI.

	Qin et al, 2023[33]	N=26 subacute IS<br>N=32 controls	DTI-ALPS	Ipsilesional ALPS was lower in subacute ischemic stroke and correlated positively with Fugl-Meyer motor scores and negatively with measures of corticospinal-tract integrity.
	Wang et al, 2025[34]	N=29 subcortical infarction<br>N=25 controls	Longitudinal 3T MRI/DTI-ALPS at 7 and 90 days	Both hemispheres showed lower ALPS than HC. ALPS correlated with MoCA at 90 days post-stroke, and the index discriminated patients with early cognitive impairment.
	Chen et al, 2025[35]	N=51 chronic stroke<br>N=27 controls	Longitudinal 3T MRI/DTI-ALPS at 3 months and 1 year	ALPS remained lower than HC at both time points; early interhemispheric asymmetry diminished by 1 year and cognition–ALPS relationships were relatively weak and time dependent.
	Yang et al, 2025[36]	N=47 CSVD<br>N=47 controls	3T MRI/DTI-ALPS	Bilateral ALPS was lower in CSVD and negatively correlated to basal-ganglia ePVS, lacunas and deep WMH.
	Toh et al, 2021[37]	N=50 ischemic stroke<br>N=44 controls	DTI-ALPS	Ipsilesional ALPS was reduced. ALPS index was associated with time since stroke.
	Zhang et al, 2021[38]	Validation study<br>N=39 CSVD<br>CSVD cohort study<br>N=330	DTI-ALPS with the use of gadolinium clearance	Modified ALPS strongly correlated with tracer-based clearance. Lower mALPS was associated with greater WMH, lacunes, microbleeds and basal-ganglia ePVS.
	Zhang et al, 2025[39]	N=31 CSVD	7T MRI/DTI-ALPS	Lower perivascular diffusivity was associated with cerebral venous disruption. DTI-ALPS index mediated the association between venous integrity and WMH and PVS volume in basal ganglia.
	Hong et al, 2024[40]	N=120; among them N=99 patients were included in longitudinal analysis	Longitudinal DTI-ALPS	Baseline DTI-ALPS was associated changes in global cognition, executive function and long-term memory. Change in DTI-ALPS predicted dementia conversion.
	Diamandi et al, 2025[41]	N=22 acute ischemic stroke<br>N=15 controls	3T MRI/DTI-ALPS	Stroke hemispheres had lower ALPS than HC. Patients receiving neither tPA nor thrombectomy had the lowest values, whereas patients receiving both treatments had the highest.
Traumatic brain injury	Zhuo et al, 2024[42]	N=44 mild TBI<br>N=37 controls	Longitudinal DTI-ALPS at ~14 days and 6–12 months post-injury	Greater acute WM-ePVS burden was associated with post-concussion symptoms/memory problems; longitudinal increases in ALPS correlated with improved sleep measures.

	Yang et al, 2024[43]	N=58 mild TBI; N=32 uncomplicated and N=26 complicated N=34 controls	3T MRI/DTI-ALPS, peak width of skeletonized mean diffusivity	mTBI showed lower ALPS and greater diffuse WM damage, particularly complicated mTBI. Lower ALPS correlated with poorer verbal memory, attention and executive function.
	Wang et al, 2024[44]	N=40 mild-to-moderate TBI; N=20 cognitive impairment, N=20 normal cognition N=20 controls	DTI-ALPS	ALPS was reduced in TBI relative to HC and was lowest in the cognitively impaired TBI cohort. Lower ALPS had strong diagnostic utility for chronic cognitive impairment.
	Park et al, 2023[45]	N=89 TBI N=34 controls	DTI-ALPS	ALPS was lower after TBI compared to healthy controls. Subarachnoid hemorrhage and diffuse axonal injury were independently correlated with lower ALPS, with a weak positive association with GCS.
	Butler et al, 2023[46]	N=37 TBI N=13 controls	DTI-ALPS, PVS volume	TBI showed modestly lower ALPS whereas lower ALPS indices negatively correlated with higher plasma NfL. PVS volume itself did not differ between groups.
	Dai et al, 2023[47]	N=161 mild TBI N=28 controls	DTI-ALPS	DTI-ALPS was in mTBI compared to controls, including MRI-negative mTBI. A weak positive correlation was further demonstrated between the ALPS index and course of disease was also observed.

Abbreviations: Aβ, amyloid-β; AD, Alzheimer's disease; ADAS-Cog11, 11-item Alzheimer's Disease Assessment Scale–Cognitive Subscale; ADNI, Alzheimer's Disease Neuroimaging Initiative; AE, autoimmune encephalitis; ALFF, amplitude of low-frequency fluctuations; ALPS, analysis along the perivascular space; ALS, amyotrophic lateral sclerosis; BOLD, blood oxygen level-dependent; bvFTD, behavioral variant frontotemporal dementia; CP, choroid plexus; CPV, choroid plexus volume; CSF, cerebrospinal fluid; CSVD, cerebral small-vessel disease; DTI, diffusion tensor imaging; DTI-ALPS, diffusion tensor imaging analysis along the perivascular space; EDSS, Expanded Disability Status Scale; ePVS, enlarged perivascular spaces; FDG, fluorodeoxyglucose; FLAIR, fluid-attenuated inversion recovery; fMRI, functional magnetic resonance imaging; FTD, frontotemporal dementia; FW-WM, free water in white matter; GCS, Glasgow Coma Scale; gMRI, glymphatic magnetic resonance imaging; iNPH, idiopathic normal-pressure hydrocephalus; iRBD, isolated/idiopathic REM sleep behavior disorder; IS, ischemic stroke; MCI, mild cognitive impairment; mALPS, modified ALPS index; MMSE, Mini-Mental State Examination; MoCA, Montreal Cognitive Assessment; MRI, magnetic resonance imaging; MS, multiple sclerosis; mTBI, mild traumatic brain injury; NfL, neurofilament light; nfvPPA, nonfluent/agrammatic variant primary progressive aphasia; PD, Parkinson's disease; PET, positron emission tomography; PLS, primary lateral sclerosis; PMS, progressive multiple sclerosis; PSCI, post-stroke cognitive impairment; PVS, perivascular spaces; RRMS, relapsing-remitting multiple sclerosis; SCD, subjective cognitive decline; sICH, spontaneous intracerebral hemorrhage; SPECT, single-photon emission computed tomography; svPPA, semantic variant primary progressive aphasia; TBI, traumatic brain injury; tPA, tissue plasminogen activator; UPDRS-III, Unified Parkinson's Disease Rating Scale, Part III; WM, white matter; WMH, white matter hyperintensities; ^{18}F-DOPA, fluorine-18 fluorodopa; ^{18}F-FDG, fluorine-18 fluorodeoxyglucose; ^{123}I-FP-CIT, iodine-123 ioflupane (^{123}I-N-ω-fluoropropyl-2β-carbomethoxy-3β-[4-iodophenyl]nortropane).

References

1. Bao C, Luo H, Wang J, Liu X, Li Y, Yang J, et al. Poor glymphatic function is associated with mild cognitive impairment and its progression to Alzheimer's disease: A DTI-ALPS study. J Prev Alzheimers Dis. 2025;12(7):100156, doi:10.1016/j.tjpad.2025.100156.
2. Kamagata K, Andica C, Takabayashi K, Saito Y, Taoka T, Nozaki H, et al. Association of MRI Indices of Glymphatic System With Amyloid Deposition and Cognition in Mild Cognitive Impairment and Alzheimer Disease. Neurology. 2022;99(24):e2648-e60, doi:10.1212/WNL.0000000000201300.
3. Li Y, Wang L, Zhong J, Xu H, Han Y, Alzheimer's Disease Neuroimaging I, et al. Impaired glymphatic function as a biomarker for subjective cognitive decline: An exploratory dual cohort study. Alzheimers Dement. 2024;20(9):6542-55, doi:10.1002/alz.14149.
4. Schirge PM, Perneczky R, Taoka T, Ruiz-Rizzo AL, Ersoezlue E, Forbrig R, et al. Perivascular space and white matter hyperintensities in Alzheimer's disease: associations with disease progression and cognitive function. Alzheimers Res Ther. 2025;17(1):62, doi:10.1186/s13195-025-01707-9.
5. Huang SY, Zhang YR, Guo Y, Du J, Ren P, Wu BS, et al. Glymphatic system dysfunction predicts amyloid deposition, neurodegeneration, and clinical progression in Alzheimer's disease. Alzheimers Dement. 2024;20(5):3251-69, doi:10.1002/alz.13789.
6. Steward CE, Venkatraman VK, Lui E, Malpas CB, Ellis KA, Cyarto EV, et al. Assessment of the DTI-ALPS Parameter Along the Perivascular Space in Older Adults at Risk of Dementia. J Neuroimaging. 2021;31(3):569-78, doi:10.1111/jon.12837.
7. Zhang X, Wang Y, Jiao B, Wang Z, Shi J, Zhang Y, et al. Glymphatic system impairment in Alzheimer's disease: associations with perivascular space volume and cognitive function. Eur Radiol. 2024;34(2):1314-23, doi:10.1007/s00330-023-10122-3.
8. Ren J, Xie D, Wang L, Wu Z, Lin S, Jin Q, et al. Glymphatic system dysfunction as a biomarker of disease progression in Parkinson's disease: neuroimaging evidence from longitudinal cohort studies. J Neurol. 2025;272(3):196, doi:10.1007/s00415-025-12944-1.
9. Zhao Y, Xu C, Chen Y, Gong T, Zhuo M, Zhao C, et al. Glymphatic dysfunction exacerbates cognitive decline by triggering cortical degeneration in Parkinson's disease: evidence from diffusion-tensor MRI. Brain Commun. 2025;7(1):fcaf029, doi:10.1093/braincomms/fcaf029.
10. Yao J, Huang T, Tian Y, Zhao H, Li R, Yin X, et al. Early detection of dopaminergic dysfunction and glymphatic system impairment in Parkinson's disease. Parkinsonism Relat Disord. 2024;127:107089, doi:10.1016/j.parkreldis.2024.107089.
11. Cai X, Chen Z, He C, Zhang P, Nie K, Qiu Y, et al. Diffusion along perivascular spaces provides evidence interlinking compromised glymphatic function with aging in Parkinson's disease. CNS Neurosci Ther. 2023;29(1):111-21, doi:10.1111/cns.13984.
12. Bae YJ, Kim JM, Choi BS, Choi JH, Ryoo N, Song YS, et al. Glymphatic function assessment in Parkinson's disease using diffusion tensor image analysis along the perivascular space. Parkinsonism Relat Disord. 2023;114:105767, doi:10.1016/j.parkreldis.2023.105767.
13. Si X, Guo T, Wang Z, Fang Y, Gu L, Cao L, et al. Neuroimaging evidence of glymphatic system dysfunction in possible REM sleep behavior disorder and Parkinson's disease. NPJ Parkinsons Dis. 2022;8(1):54, doi:10.1038/s41531-022-00316-9.
14. Zhou C, Jiang X, Guan X, Guo T, Wu J, Wu H, et al. Glymphatic system dysfunction and risk of clinical milestones in patients with Parkinson disease. Eur J Neurol. 2024;31(12):e16521, doi:10.1111/ene.16521.

15. Premi E, Diano M, Mattioli I, Altomare D, Cantoni V, Bocchetta M, et al. Impaired glymphatic system in genetic frontotemporal dementia: a GENFI study. Brain Commun. 2024;6(4):fcae185, doi:10.1093/braincomms/fcae185.
16. Jiang D, Liu L, Kong Y, Chen Z, Rosa-Neto P, Chen K, et al. Regional Glymphatic Abnormality in Behavioral Variant Frontotemporal Dementia. Ann Neurol. 2023;94(3):442-56, doi:10.1002/ana.26710.
17. Xiao D, Li J, Ren Z, Dai M, Jiang Y, Qiu T, et al. Association of cortical morphology, white matter hyperintensity, and glymphatic function in frontotemporal dementia variants. Alzheimers Dement. 2024;20(9):6045-59, doi:10.1002/alz.14158.
18. Ringstad G, Vatnehol SAS, Eide PK. Glymphatic MRI in idiopathic normal pressure hydrocephalus. Brain. 2017;140(10):2691-705, doi:10.1093/brain/awx191.
19. Ringstad G, Valnes LM, Dale AM, Pripp AH, Vatnehol SS, Emblem KE, et al. Brain-wide glymphatic enhancement and clearance in humans assessed with MRI. JCI Insight. 2018;3(13), doi:10.1172/jci.insight.121537.
20. Eide PK, Ringstad G. Delayed clearance of cerebrospinal fluid tracer from entorhinal cortex in idiopathic normal pressure hydrocephalus: A glymphatic magnetic resonance imaging study. J Cereb Blood Flow Metab. 2019;39(7):1355-68, doi:10.1177/0271678X18760974.
21. Huang NX, Zeng JY, Huang HW, Fang SY, Chen S, Li JQ, et al. Association of glymphatic system disturbance with neural dysfunction in amyotrophic lateral sclerosis. Quant Imaging Med Surg. 2025;15(4):3445-57, doi:10.21037/qims-24-1297.
22. Choi Y, Jung HJ, Jung HK, Jeong E, Kim S, Kim JY, et al. In vivo imaging markers of glymphatic dysfunction in amyotrophic lateral sclerosis: Analysis of ALPS index and choroid plexus volume. J Neurol Sci. 2025;469:123393, doi:10.1016/j.jns.2025.123393.
23. Baek SH, Tae WS, Park JW, Kim BJ. Assessment of the glymphatic dysfunction in amyotrophic lateral sclerosis using the diffusion tensor imaging along the perivascular spaces index: a pilot study. Front Aging Neurosci. 2025;17:1570327, doi:10.3389/fnagi.2025.1570327.
24. Liu S, Sun X, Ren Q, Chen Y, Dai T, Yang Y, et al. Glymphatic dysfunction in patients with early-stage amyotrophic lateral sclerosis. Brain. 2024;147(1):100-8, doi:10.1093/brain/awad274.
25. Sharkey RJ, Cortese F, Goodyear BG, Korngut LW, Jacob SM, Sharkey KA, et al. Longitudinal analysis of glymphatic function in amyotrophic lateral sclerosis and primary lateral sclerosis. Brain. 2024;147(12):4026-32, doi:10.1093/brain/awae288.
26. Carotenuto A, Cacciaguerra L, Pagani E, Preziosa P, Filippi M, Rocca MA. Glymphatic system impairment in multiple sclerosis: relation with brain damage and disability. Brain. 2022;145(8):2785-95, doi:10.1093/brain/awab454.
27. Preziosa P, Pagani E, Margoni M, Rubin M, Storelli L, Corazzolla G, et al. Glymphatic System May Mediate the Relation Between Choroid Plexus and Brain Damage in Multiple Sclerosis. Neurol Neuroimmunol Neuroinflamm. 2025;12(4):e200414, doi:10.1212/NXI.0000000000200414.
28. Bayoumi A, Hasan KM, Thomas JA, Yazdani A, Lincoln JA. Glymphatic dysfunction in multiple sclerosis and its association with disease pathology and disability. Mult Scler. 2024;30(13):1609-19, doi:10.1177/13524585241280842.
29. Zhang Y, Wu D, Fan Z, Leng X, Wan T, Shi X, et al. Abnormal glymphatic system in patients with autoimmune encephalitis: Relationship with cognitive performance. Brain Res Bull. 2025;221:111232, doi:10.1016/j.brainresbull.2025.111232.
30. Lin B, Leong YY, Mohamad M. DTI-ALPS index as a predictor of glymphatic system dysfunction in cerebral infarction. Am J Transl Res. 2025;17(6):4306-14, doi:10.62347/ANQP2661.

31. Zhang C, Sha J, Cai L, Xia Y, Li D, Zhao H, et al. Evaluation of the Glymphatic System Using the DTI-ALPS Index in Patients with Spontaneous Intracerebral Haemorrhage. Oxid Med Cell Longev. 2022;2022:2694316, doi:10.1155/2022/2694316.
32. Zhang S, Yu W, Zhang X, Cheng H, Zhang Z, Gao S, et al. Glymphatic dysfunction as a biomarker for post-stroke cognitive impairment. Sci Rep. 2025;15(1):19382, doi:10.1038/s41598-025-04054-9.
33. Qin Y, Li X, Qiao Y, Zou H, Qian Y, Li X, et al. DTI-ALPS: An MR biomarker for motor dysfunction in patients with subacute ischemic stroke. Front Neurosci. 2023;17:1132393, doi:10.3389/fnins.2023.1132393.
34. Wang Y, Yang M, Zeng X, Wang S, Zhang W, Wang W, et al. Glymphatic dysfunction assessed by DTI-ALPS index predicts early cognitive impairment in acute subcortical infarcts: a prospective clinical cohort study. Front Neurol. 2025;16:1605889, doi:10.3389/fneur.2025.1605889.
35. Chen Q, Zhong T, Liu J, Yuan B, Gao H. Chronic Glymphatic Dysfunction Modulates Domain-Specific Cognitive Recovery After Stroke: A DTI-ALPS Lesion Stratification Study. CNS Neurosci Ther. 2025;31(7):e70512, doi:10.1111/cns.70512.
36. Yang J, Liu Y, Ma Y, Zhang W, Han L, Feng H, et al. Association of glymphatic clearance function with imaging markers and risk factors of cerebral small vessel disease. J Stroke Cerebrovasc Dis. 2025;34(2):108187, doi:10.1016/j.jstrokecerebrovasdis.2024.108187.
37. Toh CH, Siow TY. Glymphatic Dysfunction in Patients With Ischemic Stroke. Front Aging Neurosci. 2021;13:756249, doi:10.3389/fnagi.2021.756249.
38. Zhang W, Zhou Y, Wang J, Gong X, Chen Z, Zhang X, et al. Glymphatic clearance function in patients with cerebral small vessel disease. Neuroimage. 2021;238:118257, doi:10.1016/j.neuroimage.2021.118257.
39. Zhang X, Pei X, Shi Y, Yang Y, Bai X, Chen T, et al. Unveiling connections between venous disruption and cerebral small vessel disease using diffusion tensor image analysis along perivascular space (DTI-ALPS): A 7-T MRI study. Int J Stroke. 2025;20(4):497-506, doi:10.1177/17474930241293966.
40. Hong H, Tozer DJ, Markus HS. Relationship of Perivascular Space Markers With Incident Dementia in Cerebral Small Vessel Disease. Stroke. 2024;55(4):1032-40, doi:10.1161/STROKEAHA.123.045857.
41. Diamandi J, Hone A, Alizadeh M, Tjoumakaris S, Gooch R, Jabbour P, et al. Evaluation of DTI-ALPS index in patients with acute ischemic stroke. Journal of NeuroInterventional Surgery. 2025;17:A47-A8.
42. Zhuo J, Raghavan P, Li J, Roys S, Njonkou Tchoquessi RL, Chen H, et al. Longitudinal assessment of glymphatic changes following mild traumatic brain injury: Insights from perivascular space burden and DTI-ALPS imaging. Front Neurol. 2024;15:1443496, doi:10.3389/fneur.2024.1443496.
43. Yang DX, Sun Z, Yu MM, Zou QQ, Li PY, Zhang JK, et al. Associations of MRI-Derived Glymphatic System Impairment With Global White Matter Damage and Cognitive Impairment in Mild Traumatic Brain Injury: A DTI-ALPS Study. J Magn Reson Imaging. 2024;59(2):639-47, doi:10.1002/jmri.28797.
44. Wang X, Deng L, Liu X, Cheng S, Zhan Y, Chen J. Relationship between glymphatic system dysfunction and cognitive impairment in patients with mild-to-moderate chronic traumatic brain injury: an analysis of the analysis along the perivascular space (ALPS) index. Quant Imaging Med Surg. 2024;14(12):9246-57, doi:10.21037/qims-24-895.
45. Park JH, Bae YJ, Kim JS, Jung WS, Choi JW, Roh TH, et al. Glymphatic system evaluation using diffusion tensor imaging in patients with traumatic brain injury. Neuroradiology. 2023;65(3):551-7, doi:10.1007/s00234-022-03073-x.

46. Butler T, Zhou L, Ozsahin I, Wang XH, Garetti J, Zetterberg H, et al. Glymphatic clearance estimated using diffusion tensor imaging along perivascular spaces is reduced after traumatic brain injury and correlates with plasma neurofilament light, a biomarker of injury severity. Brain Commun. 2023;5(3):fcad134, doi:10.1093/braincomms/fcad134.
47. Dai Z, Yang Z, Li Z, Li M, Sun H, Zhuang Z, et al. Increased glymphatic system activity in patients with mild traumatic brain injury. Front Neurol. 2023;14:1148878, doi:10.3389/fneur.2023.1148878.
